# A Randomized Controlled Pilot Trial of Different Mobile Messaging Interventions for Problem Drinking Compared to Weekly Drink Tracking

**DOI:** 10.1371/journal.pone.0167900

**Published:** 2017-02-01

**Authors:** Frederick Muench, Katherine van Stolk-Cooke, Alexis Kuerbis, Gertraud Stadler, Amit Baumel, Sijing Shao, James R. McKay, Jon Morgenstern

**Affiliations:** 1 Feinstein Institute for Medical Research, Department of Psychiatry, Northwell Health Great Neck, New York, United States of America; 2 Silberman School of Social Work, Hunter College at the City University of New York, New York, United States of America; 3 Department of Applied Health Sciences, University of Aberdeen, Aberdeen, Scotland, United Kingdom; 4 Department of Psychiatry, University of Pennsylvania, Philadelphia, Pennsylvania, United States of America; Cardiff University, UNITED KINGDOM

## Abstract

**Introduction:**

Recent evidence suggests that text messaging may help to reduce problem drinking as an extension to in-person services, but very little is known about the effectiveness of remote messaging on problem drinking as a stand-alone intervention, or how different types of messages may improve drinking outcomes in those seeking to moderate their alcohol consumption.

**Methods:**

We conducted an exploratory, single-blind randomized controlled pilot study comparing four different types of alcohol reduction-themed text messages sent daily to weekly drink self-tracking texts in order to determine their impact on drinking outcomes over a 12-week period in 152 participants (≈ 30 per group) seeking to reduce their drinking on the internet. Messaging interventions included: weekly drink self-tracking mobile assessment texts (MA), loss-framed texts (LF), gain-framed texts (GF), static tailored texts (ST), and adaptive tailored texts (TA). Poisson and least squares regressions were used to compare differences between each active messaging group and the MA control.

**Results:**

When adjusting for baseline drinking, participants in all messaging groups except GF significantly reduced the number of drinks consumed per week and the number of heavy drinking days compared to MA. Only the TA and GF groups were significantly different from MA in reducing the number of drinking days. While the TA group yielded the largest effect sizes on all outcome measures, there were no significant differences between active messaging groups on any outcome measure. 79.6% of individuals enrolled in the study wanted to continue receiving messages for an additional 12 weeks at the end of the study.

**Discussion:**

Results of this pilot study indicate that remote automated text messages delivered daily can help adult problem drinkers reduce drinking frequency and quantity significantly more than once-a-week self-tracking messages only, and that tailored adaptive texts yield the largest effect sizes across outcomes compared to MA. Larger samples are needed to understand differences between messaging interventions and to target their mechanisms of efficacy.

## Introduction

The term “problem drinker” (PD) refers to individuals who experience low to moderately severe consequences from their alcohol consumption, do not exhibit serious co-occurring drug use and/or mental health disorders, and demonstrate higher psychosocial functioning than those with severe alcoholism, but still engage in heavy drinking and experience some alcohol related consequences [1]. Most PDs never seek formal treatment and there are few treatment options for those that seek to achieve alcohol moderation vs. complete abstinence. When taken together, there is a need for new methods to reach and treat PDs. There is growing evidence that mobile short message service (SMS) is an effective, low-burden intervention platform for a broad range of physical and mental health problems [2]. Studies across health behaviors reveal that SMS is more effective than treatment-as-usual without SMS, and patients tend to prefer SMS to other delivery mediums such as phone calls or email [2–5].

SMS interventions are emerging as a promising medium to deliver substance abuse-specific health interventions, as they are intrinsically proactive and can be accessed by the user at any time. Thus, goal-salient messages can be delivered at critical moments in substance abuse trajectories, such as when a craving occurs [6]. In the earliest mobile messaging study, Weitzel and colleagues [7] sent college students alerts about the consequences of drinking via a stand-alone personal digital assistant. The program yielded a significant short-term reduction in drinks per drinking day compared to assessment-only over a two-week period. More recent studies have examined the impact of text messages delivered on the weekends to non-treatment seeking young adults released from the ED [8], daily motivational messaging delivered to college students [9], and messages to address co-morbid alcohol use and depression [10].

There have also been some promising studies using smartphone applications for PD [11, 12] and for more severe drinkers as an aftercare intervention following treatment [13]. However, smartphone applications and internet interventions should not be considered equivalent to SMS interventions simply because they can all be viewed on the mobile phone. Each has their strengths and weaknesses. For example, SMS is delivered proactively without user effort, while a mobile app has to be opened by the user. By contrast, mobile apps can deliver engaging graphics and collect passive information, which SMS is not designed to do. This paper specifically describes the impact of SMS on problem drinking.

Despite the recent literature on SMS for PD and other behavioral health problems, very little is known about the mechanisms that may explain the promising outcomes of SMS interventions, such as the impact of tailored message content, optimized timing of SMS delivery, and theory-driven vs. general motivational message content [4]. In their meta-analysis of SMS interventions for health, Head and colleagues [5] revealed that SMS interventions can vary dramatically across protocols in terms of factors such as which behavior change theory (if any) is driving message content, SMS frequency and timing, user interactivity, and the type and degree of content tailoring to the individual user. Understanding how variations in these factors impact PD is an important step towards building more effective mobile interventions. To our knowledge, there have been no studies directly comparing many of these factors in a single mobile messaging study.

### Types of Tailoring

Gain and loss-framed message content has received significant attention in the general health promotion literature and is the most studied framing paradigm for digital interventions across health domains [4, 14, 15]. Overall, these studies indicate that gain-framed messages modestly outperform loss-framed messages, but differential outcomes are often moderated by factors such as readiness to change or the target health behavior. However, message framing has not been examined specifically when delivered via text messaging.

Similarly, while message tailoring has been studied in the general health promotion literature, there has been virtually no direct examination or comparison of tailored vs. untailored mobile interventions for any behavior. Tailoring can refer to personal relevance (e.g. name) or tailoring to a specific target behavior-related characteristic (e.g. severity, self-efficacy). Studies have generally suggested that content-tailored interventions yield better outcomes than untailored interventions, especially when content of the untailored intervention has no relevance to the recipient’s personal experiences [16–18]. An additional form of tailoring entails adapting the intervention content to the current needs of the individual, which we refer to as adaptive tailoring. While the literature on comparing adaptively tailored interventions to statically tailored interventions is lacking, some research has shown that tailored computer-based interventions that adapt to the recipient’s progress (or lack thereof) outperform static tailored interventions [18].

The primary aim of this pilot study was to advance the literature on SMS interventions for PD by examining the effects of gain-framed (GF), loss-framed (LF), statically tailored (ST) and adaptively tailored (TA) SMS programs compared to weekly mobile assessment only (MA) on alcoholic drink reduction over a 12-week period in individuals seeking assistance on the internet to reduce their alcohol consumption to safe levels. To achieve these aims, we conducted an exploratory, single-blind, randomized controlled pilot study comparing effect sizes of weekly self-tracking mobile assessment only to the different daily messaging groups on reductions in weekly drinks, weekly heavy drinking days, number of days without drinking and drinking consequences over a 12-week period. Outcomes are designed to generate inform the development and testing of future messaging studies to reduce problem drinking.

## Method

### Participants and Procedure

Participants were recruited between April 2014 and January 2015 through online alcohol screening and help-seeking sources, such as AlcoholScreening.org and Moderation.org. Advertisements offered individuals worried about their drinking the opportunity to screen for a research study to find out if texts could help them manage their alcohol consumption. Prospective participants were directed to the study website, which offered basic information about the study and a link to a brief screening survey in Survey Monkey. IP blocking once the web screening was completed ensured that participants could only complete the survey once from any given device. Individuals eligible to continue the screening process based on the survey were provided with the study’s email address to schedule a time to complete a 20-minute phone screening with study staff. Participants deemed eligible based on the screening call provided verbal assent and were emailed two links: one to a web-based consent form and consent form quiz, and another to the baseline survey. All participants electronically signed the consent form and any incorrect answers to the consent form quiz were reviewed to ensure comprehension. Ineligible participants were directed to online drinking resources, the SAMHSA Treatment Finder, and were offered referrals.

Participants were eligible for the study if they: (1) consumed at least 13 and 15 standard drinks per week for women and men, respectively; (2) were willing to reduce their drinking to non-hazardous levels by study completion; (3) were between the ages of 21–65; (4) owned a mobile phone and were willing to receive and respond to text messages; (5) were fluent in English; and (6) could read at the 8th grade level, as determined by the consent form quiz. Participants were excluded from the study if they: (1) drank more than 45 standard drinks per week; (2) demonstrated clinically severe alcoholism (evidenced by physical withdrawal symptoms or a history of serious withdrawal symptoms, such as hallucinations or seizures); (3) scored above a 12 on the Short Alcohol Withdrawal Scale [19]; (4) presented with a current substance use disorder for any substance other than alcohol, nicotine, or caffeine (defined as more than once weekly use in the past month); (5) used marijuana more than twice weekly in the past month; (6) self-reported or presented with a serious psychiatric illness as evidenced by a previous diagnosis of psychosis or bipolar disorder, inpatient treatment, medication for psychosis, or recent suicidality; (6) were already in alcohol treatment or expressed a desire to obtain additional substance abuse treatment while in the study; (7) reported a medical condition that precluded drinking any alcohol, pregnancy or a desire to become pregnant while in the study; (8) reported a desire to pursue either trial or long-term abstinence; or (9) demonstrated a lack of understanding of study protocol or reading difficulty as evidenced by a score of less than 7 out of 10 on the consent form quiz. The majority of eligibility criteria were assessed during the online self-report screening questionnaire. About halfway through the trial, we reduced the drinking criteria for eligibility from 21 and 24 standard drinks per week for women and men, respectively, to 13 for women and 15 for men due to the number of drinkers screened who did not meet the original threshold, but still reported problems resulting from their drinking. There were no differences at baseline in drinking severity between messaging groups. This study was approved by both the New York State Psychiatric Institute Institutional Review Board (IRB) and the Northwell Health Institutional Review Board (IRB) (S1 Fig), and all clinical investigation was conducted according to the principles expressed in the Declaration of Helsinki.

**Fig 1:**
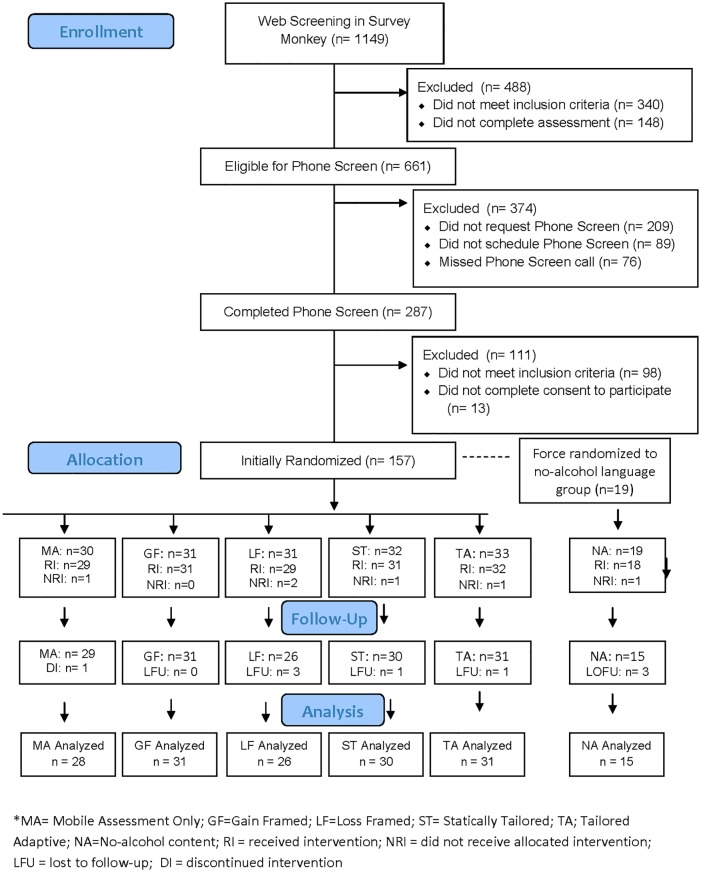
CONSORT 2010 flow diagram.

**Fig 2:**
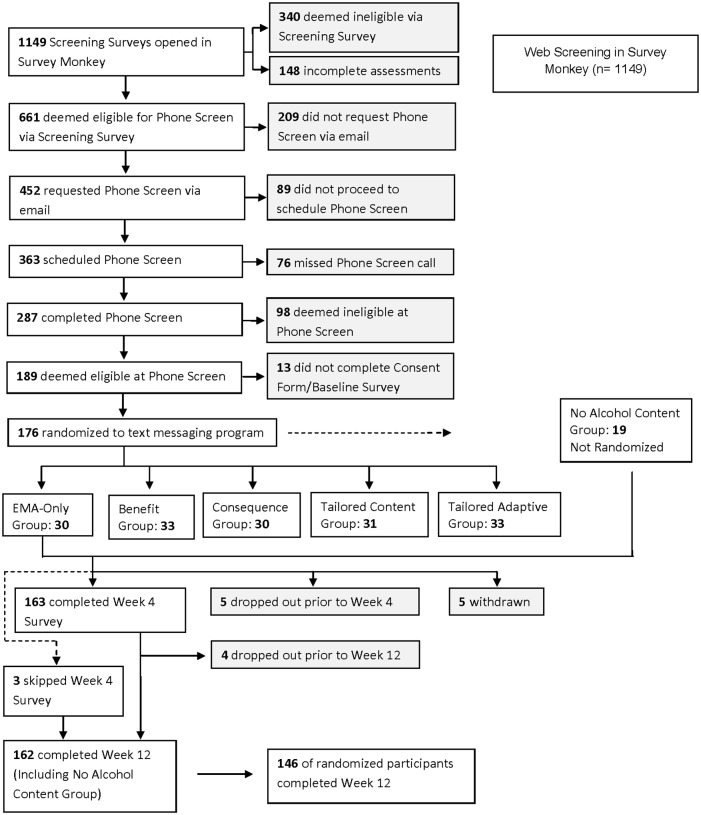
Participant flow.

Study flow, reasons for exclusion, study assignment, and retention are presented in the Consort Flow Diagram (Fig 1). Within an 8-month period, 1,149 individuals completed the screening questionnaire and 661 were eligible for phone screening based on the initial web-screen. Of those, 287 completed a phone screening. In total, 189 individuals were deemed eligible for study participation based on the web- and phone- screenings, and 176 completed the consent form and baseline assessment. 19 participants were force-randomized into a group with no specific alcohol content based on their request to receive messages that did not include terms referring to alcohol and drinking. Because this small group was force-randomized, they are presented separately from the active messaging groups. We present the descriptive findings for this group in the results section. Of the remainder, 157 were randomized to a study condition after completing the consenting process and five were withdrawn within the first week of randomization. Of the five participants excluded from the final sample, one was excluded due to incoherent and inconsistent responses to baseline web-based assessments, one was withdrawn due to pregnancy, one was excluded due to a technology-based error that precluded the user from receiving the text messages, one was excluded for drinking too little at baseline to be included in analyses, and one was excluded as a “technology troll” (someone working for a company developing mobile apps for health behavior change) who mined the program’s content for a week before dropping out. Of the remaining 152 participants who completed the baseline assessment and were not withdrawn, seven were lost to contact prior to the week 12 assessment. The final randomized sample included 152 participants at baseline and 145 participants at week 12. Of the 19 participants force-randomized into the no-alcohol content group, 17 completed their week 12 assessment. See Fig 2 for details. The trial ended when at least 30 participants were enrolled in each group at baseline to ensure adequate power to estimate stable effect sizes which stabilize at approximately 30 participants per group [20].

#### Procedures

Once the participant completed the baseline survey, they were randomized to one of the five study conditions (see below) by the project research assistant (RA), stratified by gender and alcohol consumption. Envelopes were created based on gender (male/female) and high and low drinking, with equal chances of being selected into groups. At the halfway point of the trial, we checked group allocation and weighted the envelopes to create equal allocation across conditions. Once randomized, the RA assigned the participant into the proper messaging group, and all participants were emailed a *What to Expect* document that corresponded to their study condition. Participants were blind to the study condition and were only aware that the type and number of messages differed between conditions. The condition-specific document trained participants on how to respond to the weekly mobile assessment (MA) texts and provided them with NIAAA guidelines for safe drinking by gender and information on what constitutes a standard drink [21]. Participants started to receive text messages within one business day of being randomized to a condition. All participants received a welcome text message within a few minutes of their program’s activation, which provided instructions on what to text if they experienced any problems with their program. At week 4, the RA emailed the participant a link to a brief check-in survey to monitor their progress and user experience and offered the participant the opportunity to schedule a phone check-in with the study PI. If, after prompting, the individual did not complete the week 4 web assessment, the RA waited to reach out to them again until week 12. At week 12, the RA emailed the participant a link to their final web-based assessment. Upon survey completion, participants were offered the choice of a $40 Amazon or Starbucks gift card and the opportunity to receive an additional 12 weeks of the text messaging program of their choice. We ended enrollment when at least 30 participants were enrolled in each group.

#### Assessments

The self-report internet screen included items to screen out potential participants based on age, drinking quantity and frequency via the QFV-30, a 30-day shortened time frame version of the Form-90 Quantity-Frequency measure of alcohol consumption [22], which has been validated against the Timeline Follow-Back [23], alcohol withdrawal and severity via the Short Alcohol Withdrawal Scale [19], and drug use via the 30-day frequency scale of the Addiction Severity Index [24]. The phone screen included a brief assessment of both substance use and mental health treatment history, current substance use quantity and frequency if indicated in the screener, and a brief diagnostic screening for Bipolar Disorder and Psychosis using the SCID-IV stem questions [25]. Finally, we collected location and collateral information in case of an emergency.

At baseline and week 12, we assessed drinking outcomes with a more detailed version of the QFV-90, asking participants to provide an estimate of their average alcohol consumption per week and then an estimate of their average consumption by weekday (e.g. You drink about 25 drinks per week. Using this average, about how many drinks do you have on Monday, Tuesday, etc.). For participants randomized to the tailored adaptive group, we used responses to this item to send personalized texts on their heaviest drinking days. We assessed drinking consequences via the Short Inventory of Problems (SIP) [26]. All measures were repeated at week 12 with additional questions on subjective drinking outcomes, satisfaction and program preferences. A brief 5-minute survey using the QFV-30 and self-report subjective goal achievement questions was conducted at week 4 to understand short-term changes in drinking.

### Study Interventions

Participants in all conditions were given the NIAAA guidelines for safe drinking and asked to follow these guidelines to ensure that all participants were aware of the safe drinking guidelines and to offer more credibility to the mobile assessment control, as have been used in previous web-based interventions for alcohol use problems [27]. Participants were led to understand that their messaging program would differ from the others with regard to content and frequency.

#### Mobile Assessment only (MA)

The MA group was designed to be a self-monitoring control, which has been used in both general health based SMS studies [2] and alcohol treatment studies as an intervention and control [28]. Participants in this condition were informed that they were in a drink-tracking program and received four questions about the past week’s drinking once weekly. If participants did not respond to the first text within a half hour, the system resent the text up to three more times over the course of the next two hours. If participants did respond to the first text, the system would then send the second question. Participants in *all* messaging conditions received the MA as their base program.

#### Loss-Framed messaging (LF)

The loss-framed messaging program included text messages sent at 6:00 PM daily on the consequences of problem drinking. Messages were based on the existing literature and used both the social learning theory and the health belief model [29], which highlight that the short- and long-term consequences of heavy drinking can motivate behavior change. Loss framing is a primary component of many SMS interventions, including those that have targeted problem drinking [7]. An example of an LF message is, “Think of all you have lost as a result of drinking too much. Make today a day that sets the stage for change.”

#### Gain-Framed messaging (GF)

The gain-framed messaging program includes text messages sent at 6:00 PM daily on the benefits of reducing drinking to safe guidelines. Messages were mirrored to the LF program to provide information on the benefit that most corresponded to the opposite behavior described in each LF text message. This was also based on the social learning theory and the health belief model, with a focus on the benefits of not engaging in heavy drinking rather than the consequences of heavy drinking. An example of a GF message is, “Think of all you can achieve if you can control your drinking. Make today a day that sets the stage for change.”

#### Statically Tailored content (ST)

The ST treatment arm included tailored text messages sent at 6:00 PM daily based on individual responses to the baseline assessment. Tailoring included tailoring to the day of the week (e.g. a specific message on Friday night referencing the weekend), tailoring to the day in the program (e.g. “It’s been three weeks since you’ve signed up…”), and approximately 50% of messages were tailored based on participant gender, age, binge vs. daily drinking habits, solitary vs. social drinking habits, consumption severity, self-efficacy, motivation, craving, predicted effort required to reduce, and acceptance of the need to reduce alcohol consumption. The tailoring algorithm for continuous variables differed based on the median from our previous studies with problem drinkers.

#### Tailored Adaptive (TA)

The TA intervention included all ST features along with three additional components. First, messages individuals received over the course of a week varied based on their goal achievement in the prior week. Second, two additional messages were sent that included the TA participant’s name each week at their heaviest typical drinking times. Third, participants were able to proactively text the automated system key words in order to receive just-in-time support, followed by a number of text-based check-ins. Key words included: *tempt* for support to manage a craving to drink, *drink* for support when the participant had started drinking, *heavy* for support when the participant had started drinking heavily, *win* for support either when the participant succeeded at managing a craving or when the participant succeeded in drinking no more than their moderate drinking goal for the situation, and *regret* for support after failing to moderate.

#### Non-Alcohol content motivational messaging (NA)

An additional messaging program was included for participants who wanted to receive text messages that did not contain any reference to alcohol for reasons of stigma and privacy. Text messages were sent at 6:00 PM daily and included motivational messages on achieving one’s goals, the benefits of change, and the consequences of not changing. Mobile assessment in this group substituted the word *coffee* for *alcohol*. Messages were mirrored to the LF and GF programs as much as possible. The goal of including this program was to understand how many people would opt for NA messages and whether there would be a difference between NA and targeted content groups in terms of outcomes. This group was not included in primary analyses.

### Data Analysis

As in previous drinking moderation studies [1], the two primary outcomes were weekly sum of standard drinks (SSD) and weekly sum of heavy drinking days (HDD). These were selected a priori because NIAAA safe drinking guidelines include measures of drinking quantity and intensity. Secondary outcome measures included number of days without drinking per week and consequences of heavy drinking, as measured by the SIP. Both intent-to-treat and completer analyses were performed. To determine whether differential attrition occurred by condition, we investigated whether group differences existed for baseline scores and rates of follow-up at each assessment point. Results indicated no significant differences in attrition between conditions and no significant baseline differences in socio-demographic and alcohol severity variables between those lost to follow-up and those who completed their 12-week program. To analyze the three count outcomes (number of drinks per week, number of heavy drinking days per week, number of days per week per week without drinking), we used Poisson regression models including a scaling parameter (*ϕ*) to allow for overdispersion and for non-normal distribution of count data [30]. For the continuous outcome (consequences), we conducted ordinary least squares regressions. In all these regression models, we first conducted regressions controlling for baseline values comparing TA to MA on all primary outcome variables to test our primary hypothesis that TA would be superior to MA on all drinking outcomes. We further conducted regression analyses for all outcomes by group, with four dummy-coded indicators comparing each of the four intervention groups with the control group (1 = intervention group, 0 = control group), adjusting for baseline centered at the grand mean. The sample size enabled a test of the secondary hypotheses of a medium effect size at power = .80 when comparing differences between the individual active treatments and the control. We conducted all analyses using IBM SPSS 23 with a significance level set to .05.

## Results

### Demographics and Mobile Phone Characteristics

Descriptive data for the sample are presented in Table 1. Participants had a mean age of 43.2 (SD = 11.1), and 74.9% were female. The sample was racially and ethnically homogeneous, with 93.5% identifying as white and only 2.4% reporting Hispanic/Latino ethnicity. One hundred and thirty-five participants (79.9%) had owned only one personal cell phone in the last year, and 95.9% reported that they had never had their service turned off for any reason. With regard to text messaging plans, 90% had unlimited SMS and 9.4% a monthly limit. Overall, most participants (80%) sent and received between 0–100 messages per month while 16.5% received between 101–500 messages per month. At baseline, participants were drinking 24.9 (8.4) standard drinks (SDs) per week on average, drinking on 5.2 (1.2) days per week on average, and drinking four or more drinks in a single sitting on 3.4 (2.3) days per week. Participants’ drinking goals skewed towards wanting to reduce both the amount of alcohol consumed per day and number of days drinking per week (78.3%), with 5.1% wanting to reduce the number of days they drank only and another 5.1% wanting to reduce the amount of alcohol consumed per day only. Of those who did not specify any of these goals, 9.4% reported wanting to reduce in general. There were no significant differences between groups at baseline on primary outcome variables. Participants responded to 89.1% of weekly self-tracking, ranging from a low of 74% in the LF group to a high of 97% in the TS group.

**Table 1:** Demographics.

Variable	Answer	N = 172%
Gender	Female	128 (74.9%)
Race	Black	2 (1.2%)
	White	157 (93.5%)
	Asian	2 (1.2%)
	Other	7 (4.2%)
Ethnicity	Hispanic	4 (2.4%)
Text Messaging Plan	Unlimited	153 (90%)
	200 messages a month	16 (9.4)
	Per message	1 (.6)
Texts sent/received per week	1–100	136 (80)
	101–500	28 (16.5)
	>500	6 (3.5)

Note: All participants, including those in the NA group are represented above. There were no significant differences between groups on any demographic variables.

### Weekly Sum of Standard Drinks (SSD)

**Fig 3:**
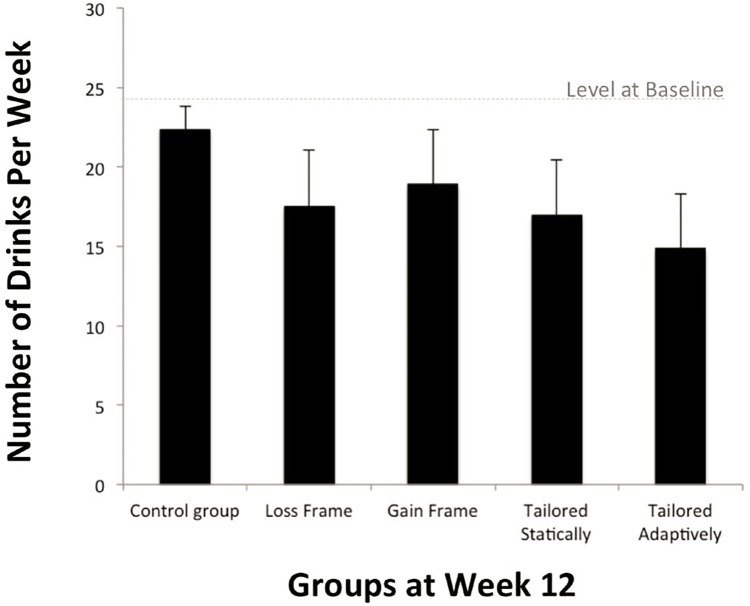
Estimated means and standard deviations of number of drinks per week.

Please see Table 2 for descriptive statistics on all primary outcomes at baseline and week 12, and Table 3 for results of the regression models. Weekly sum of standard drinks (SDD) in the 30 days prior to the week 12 assessment revealed that participants in all treatment groups reduced their weekly alcohol consumption more than participants in the control group except GF (statistical trend, *p <* .*09*.), with the TA group yielding the largest effects, *b = -0*.*40*, *p <* .*001*. Multiple comparisons between treatment groups indicated that no treatment group emerged as significantly better than others, as indicated by overlapping confidence intervals. Changes from the baseline to week 12 by group are presented in Fig 3.

**Table 2:** Baseline and Week 12 Drinking Outcomes.

	Nr. of drinks per week				Nr. of heavy drinking days per week				Nr. of days per week without drinking				Consequences				% Moderate/ Significant Improvement
	Baseline		Week 12		Baseline		Week 12		Baseline		Week 12		Baseline		Week 12		
Groups	*M*	*SD*	*M*	*SD*	*M*	*SD*	*M*	*SD*	*M*	*SD*	*M*	*SD*	*M*	*SD*	*M*	*SD*	
Control Group	24.69	9.81	22.00	9.65	3.03	2.53	2.89	2.48	0.41	0.82	0.68	1.02	1.84	0.42	1.77	0.50	17.9%
Loss Frame	25.52	6.57	18.00	9.72	3.76	2.28	2.27	2.44	0.69	1.31	1.50	1.77	1.87	0.53	1.65	0.43	34.6%
Gain Frame	24.77	9.73	19.03	10.12	3.23	2.26	2.29	1.95	1.16	1.42	1.90	1.80	1.92	0.50	1.75	0.64	41.9%
Tailored Statically	24.55	7.86	17.10	7.56	3.26	2.44	1.83	2.00	0.61	0.88	1.43	1.33	1.80	0.31	1.59	0.32	46.7%
Tailored Adaptively	24.16	6.46	14.52	5.80	3.69	2.13	1.53	1.43	1.00	1.24	1.90	1.58	1.78	0.33	1.56	0.37	51.6%

**Table 3:** Comparison of drinking in the four intervention groups (Loss Frame, Gain Frame, Tailored Statically, Tailored Adaptively) with the control group at Week 12: Estimated means and standard errors from residualized regressions, adjusting for baseline levels.

	Nr. of drinks per week^a^				Nr. of heavy drinking days per week^b^				Nr. of days per week without drinking^b^				Consequences^b^		
Regression type	Poisson				Poisson				Poisson				OLS		
	*b*	*SE*	*RR*	*p*	*b*	*SE*	*RR*	*p*	*b*	*SE*	*RR*	*p*	*b*	*SE*	*p*
Intercept	3.09	0.07	21.87	0.00	0.99	0.14	2.69	0.00	-0.31	0.28	0.73	0.27	1.77	0.07	0.00
Baseline	0.03	0.004	1.03	0.00	0.24	0.03	1.27	0.00	0.32	0.06	1.38	0.00	0.68	0.07	0.00
Loss Frame	-0.23	0.11	0.79	0.03	-0.41	0.21	0.66	0.05	0.60	0.35	1.83	0.08	-0.14	0.10	0.16
Gain Frame	-0.18	0.10	0.84	0.09	-0.29	0.20	0.75	0.15	0.72	0.33	2.06	0.03	-0.07	0.09	0.45
Tailored Statically	-0.27	0.11	0.76	0.01	-0.55	0.22	0.58	0.01	0.70	0.34	2.02	0.04	-0.15	0.09	0.12
Tailored Adaptively	-0.40	0.11	0.67	0.00	-0.72	0.23	0.49	0.00	0.78	0.33	2.18	0.02	-0.17	0.09	0.07
Scaling parameter *φ*	3.22				1.53				1.51				N/A		

*Note*. OLS = ordinary least squares. Baseline was centered at the grand mean in the sample, and all other predictors are dummy-coded comparisons of each intervention group with the assessment-only control group. Thus, the intercept indicates the average outcome in the control group for a participant with average baseline drinking levels in the sample. The other regression coefficients indicate how each intervention group differs from the control group.

^a^*N* = 146.

^b^*N* = 145.

### Heavy Drinking Days

Assessment of the weekly sum of heavy drinking days (HDD) revealed a similar pattern, such that a significant main effect was observed for all treatment groups except GF (*p* =.*15*) when compared to MA. As with SSD, the TA group yielded the largest effects on this measure, *b = -0*.*72*, *p <* .*0001*. As before, multiple comparisons between treatment groups indicated that no treatment group emerged as significantly better than others, as indicated by overlapping confidence intervals.

### Days without Drinking

All treatment groups increased the number of days without drinking over the course of the trial, except LF (*p* = .*08*) when compared to MA, again with the strongest effect in the TA group, *b = 0*.*78*, *p* = .*02*. As before, multiple comparisons between treatment groups indicated that no treatment group emerged as significantly better than others, as indicated by overlapping confidence intervals.

### Consequences

There were no significant reductions in the type or severity of consequences as measured by the SIP over the course of the trial compared to MA, although there was a statistical trend towards consequence reduction in the TA group, *b = -0*.*17*, *p = 0*.*07*. Post-hoc analyses revealed that those who reduced their drinking to safe levels had significantly lower SIP scores at week 12 than those who did not, *F = 5*.*16*, *p* = .*024*.

### Subjective Goal Achievement

All groups reported significant subjective improvements in their drinking behavior from baseline to week 12 when compared to MA. The percent of participants in each group who reported at least moderate improvement in drinking over the course of the 12 weeks was as follows: 17.9% MA, 34.6% LF, 41.9% GF, 46.7% ST, 51.6% TA, and 31.3% NA. The partial correlation between subjective overall change and actual change in SDD was significant, *r* = .*49*, *p <* .*0001*. Finally, 79.6% of all participants assessed at week 12 expressed a desire to continue receiving text messages for an additional 12 weeks. However, only 64.4% of the total sample reached out to the research assistant to select and enroll in a new program.

### Descriptive Assessment of Non-Alcohol Messaging Group

Overall, the NA group reduced their SSD by over 5.5 drinks per week as compared to 2.5 in the MA group. When examined separately, the weekly drink reduction was similar to that observed in the GF and LF groups, but the effects were smaller and insignificant for both HDD (.54 day decrease) and days without drinking (.66 day increase), when compared to MA. Dichotomous analyses indicate that 31.3% reported at least moderate improvement in their drinking, which is similar to the LF group (34.6%).

## Discussion

This is the first study to test theoretically distinct text messaging programs for any behavior change goal or health condition to weekly self-tracking assessment texts. All active messaging groups, except GF, were significantly superior to MA in reducing weekly alcohol consumption and heavy drinking days. Only the TA and GF groups were significantly superior to MA in reducing the number of drinking days per week. While TA had the largest effect sizes compared to MA, there were no significant differences between active messaging groups.

Within an 8-month period, 1,149 individuals completed the screening questionnaire and 661 were eligible for the study, suggesting remote messaging interventions are an acceptable and feasible means to engage a large number of help-seeking PDs. Yet another notable finding was the remarkably high rate of participant retention from baseline to week 12 (94%) and the high level of interest expressed by participants to continue receiving messages after the treatment period. While only 64.4% of participants who completed the week 12 assessment actually signed-up, these findings are in contrast with the attrition rates associated with mobile app- and web-based digital interventions. SMS is an inherently proactive intervention, as messages automatically appear on the users’ screens, while web-pages and apps require user initiation. Thus, texting requires less user effort for the participant to receive intervention content reducing burden on the client to take the minimal necessary action to change.

The primary reason for ineligibility at screening was low weekly alcohol consumption but a consistent pattern of single-night binge drinking. Given the often severe consequences of binge drinking, this group should be included in future trials. Similarly, we lost numerous potentially eligible participants between the web-screen and phone screening, suggesting a need for new methods to keep these individuals engaged in the enrollment process. Of the individuals not eligible during the phone screening, the majority were taking other substances regularly, including prescription benzodiazepines.

When examined individually, all messaging groups outperformed MA on most primary outcomes. Like other messaging interventions for drinking with non-treatment seeking samples [8], the greatest effects were observed for the reduction in the number heavy drinking days, rather than increasing the number of days without drinking more generally. It is possible that messaging may be particularly helpful in assisting in self-regulation of drinking episode intensity to a greater extent than in assisting individuals to abstain more often.

### Active Messaging Conditions

This study was not powered to detect significant differences between active groups, but the findings can inform future messaging development for health behaviors. Notably, TA consistently had the largest effect sizes among treatment groups, although effect sizes amongst treatment groups did not significantly differ. All messaging groups yielded meaningful reduction in weekly drinking, which highlights that even simple untailored daily messaging can have a positive impact on alcohol-related behavior. That these automated mobile programs significantly reduced heavy drinking has implications for improving the provision of low burden resources to heavy drinking help-seekers.

The larger effect sizes, albeit not significant, of the TA group compared to others adds support for further study of just-in-time adaptive interventions to enhance behavioral outcomes [31, 32]. However, the mechanisms to explain larger effect sizes of the TA intervention could not be determined in the current study. It could be that the additional messages individuals received on their heaviest drinking nights, the “just-in-time” messaging feature, the addition of the participants’ names in several messages, or the adaptive nature of the intervention to the users’ goal achievement from week to week differentially impacted outcomes. It is also possible that the increased quantity of messages received by the TA group accounted for the difference in outcomes, as it has been posited that the benefit of tailoring may actually be in the increased contact associated with most tailored interventions compared to untailored interventions [18]. More research is needed to dismantle the effects of these interventions and understand the mechanisms of efficacy. Fig 3 highlights the descriptive change from baseline to week 12. While no strong conclusions can be made based on this pilot data, these preliminary results are consistent with the larger literature suggesting the benefits of tailoring and adaptation, and should provide guidance for future studies.

### Gain and Loss Framing

This was the first text messaging study to compare gain-framed vs. loss-framed mobile messaging for any behavior. We were surprised to find few differences in outcomes between these groups on any measure. While there were slight differences in the effect sizes of these groups compared to MA, they were not significant. Historically, GF and LF have been differentially used such that LF is used to motivate individuals to avoid future problems and GF to facilitate progress [33]. Future research on messaging for PD should examine moderators of gain- and loss-framing used in the health promotion literature in general, such as motivation style, severity and locus of control [15, 17, 34]. In our work developing an SMS intervention for addiction continuing care, we found that while most participants preferred GF messages, those individuals who preferred LF messages perceived fewer overall benefits of changing and had lower readiness to change scores. Understanding these differences in preferences as individuals navigate the change process is an important undertaking and will be explored in later papers.

### Other Outcomes

There were no significant reductions in drinking consequences for any of the treatment groups compared to MA, though the TA group’s consequence reductions trended towards significance. This is similar to the findings of previous studies on brief in-person and web-based interventions which revealed few meaningful reductions in alcohol consequences [27, 35, 36]. It is possible that the reductions in drinking quantity or frequency did not translate into significant reductions in consequences. Those who reduced their drinking to safe levels had significantly lower SIP scores at week 12 than those who did not, suggesting the ability of the measure to discriminate between heavier and lighter drinkers. Future research will be needed to understand this discrepancy. It is also possible that the SIP measure may be more appropriate in abstinence-focused studies and that changes in some consequences may not be readily apparent early in the change process of those pursuing moderation goals. Longer-term follow-up assessments are needed to test this.

The addition of the general motivational messaging program containing no reference to alcohol represents important ground work for understanding the differential impact of content specificity vs. non-specificity on behavior change attempts. Although this group was not randomly assigned and therefore results should be viewed as exploratory, the NA group had smaller effect sizes on most outcomes than those of all other active messaging groups. This finding is in line with recent research suggesting that messaging targeted to the presenting problem was more effective in improving health outcomes than non-targeted messaging [4]; however, this finding warrants further study, as general non behavior-specific messaging may be used to circumvent some of the safety and security regulations that currently limit mHealth treatments. HIPAA regulations require not only secure messaging options, but also creative alternatives that help us integrate messaging into care in a cost effective manner. It is important to note that, like the MA group, it appears the NA group may not be as robust over time as the other groups.

While this treatment development and pilot study provide a rationale and foundation for further study of remote mobile SMS as an intervention for problem drinking, several limitations exist. First, sample sizes for each condition were small, limiting power for analyzing multiple treatment groups. Our sample was predominately white and female, limiting generalizability of findings to more diverse samples. The sample enrolled was similar in terms of gender break-down to the 1,149 total individuals screened on the web, suggesting that SMS may be particularly attractive to women seeking help or information online. This study was also very conservative with regard to who was enrolled compared to open real-world, web-based intervention studies, limiting generalizability to the heterogeneous group of PDs that might seek help on the internet. Expanding the reach of future studies to include a larger subset of treatment-seekers might result in significantly more individuals receiving messaging programs. Similarly, future research with larger samples will be needed to test effects over time before long-term efficacy can be determined.

This study utilized consumption assessment methods that at present are not the gold standard metric when assessing in-person alcohol consumption (e.g. Timeline Follow-Back or objective validation of results). Future research with more resources should compare the reliability and validity of these brief measures when completed remotely with validated measures such as the online Timeline Follow-Back [37] or transdermal alcohol sensing. However, there is a robust web-based intervention literature using similar assessment methods, and the relative change between study conditions provides a useful comparison of messaging impact between groups [27, 38]. Despite these limitations, this study provides further evidence for the utility of SMS as a tool to reduce alcohol consumption remotely in a heavy drinking sample. Future research will need to test effects over time before long-term efficacy can be determined.

## Supporting Information

S1 Fig SMSProtocolMuench. (PDF)

S2 Fig SMSMuenchconsortchecklist. (PDF)
