# Supplementary material for: A Randomized Controlled Pilot Trial of Different Mobile Messaging Interventions for Problem Drinking Compared to Weekly Drink Tracking
Source: PLoS One. 2017 Feb 1;12(2):e0167900. doi: 10.1371/journal.pone.0167900 (PMC5287456; doi:10.1371/journal.pone.0167900)
Supplement: S1 Fig — (PDF) [file pone.0167900.s001.pdf]

Protocol

Tailored Mobile Text Messaging to Reduce Problem Drinking

Funded by NIAAA R34 AA 021502
National Institute of Alcohol Abuse and Alcoholism

Frederick Muench, Ph.D.

(Note: This study was recruiting under NYSPI/RFMH IRB approval (6625) and is being transferred to NSLIJ. Do not have NSLIJ contact information yet).

fmuench@nshs.edu

5/30/2014
Revised
7/16/2014

INTRODUCTION/BACKGROUND MATERIAL/PRELIMINARY STUDIES AND SIGNIFICANCE

Despite the new growth of SMS interventions and early promising studies in other areas, there have been no published SMS studies with problem drinkers looking to change their drinking, and to our knowledge there have been no SMS studies across any health problem or disorder has directly compared tailored vs. untailored messaging and the effects of dynamically timed-interactive messaging vs. statically timed messaging outcomes. As a recent NIH review of mobile health interventions highlights, we need to test new behavior change models with the advent of mobile technologies and incorporate the adaptive capabilities into our treatments (Riley, Rivera et al., 2011). Our previous work on the feasibility of using text messages with disenfranchised individuals in addiction treatment revealed that 98% are interested in using SMS to help them stay drug free and that individuals preferred different types of messaging at different periods of recovery.

According to the National Institute on Alcohol Abuse and Alcoholism, problem or risky drinking is defined as greater than 7 standard drinks per week for women and 14 standard drinks per week for men. Other groups have other criteria (e.g. 10 drinks for women and 14 for men per week). The Institute of Medicine reports that problem drinkers are those with mild-to-moderate problem severity who do not have physical dependence. These individuals are not severe enough to meet criteria for tolerance and withdrawal but still report trouble controlling their drinking. These individuals are not likely to seek treatment but are interested in methods to reduce their drinking.

The large literature from other areas reveals that SMS interventions offer several unique benefits that appear generalizable to warranting their examination with individuals in PD. First, messaging interventions inherit the benefits of internet interventions (e.g. low cost, standardized, individualized, adaptable and tailored, allow for data collection) but there is no need for an internet connection, or to use a computer following the initial assessment (or ever). Second, unlike mobile apps, SMS interventions do not require a smart phone, custom software programming for specific phones, application updates, and are accessible on most phones making the intervention significantly more accessible and generalizable than smart phone apps. Third, SMS ready phones can perform nearly all the functions of standalone ecological momentary assessment (EMA) devices without additional hardware or custom programming. There is an emerging literature on the using of text messaging to capture EMA data and results reveal similar outcomes to PDAs and IVR (Berkman, Dickenson, et al., 2011). One great advantage of SMS is it does not require the person to be immediately present like interactive voice response EMA.

The study entitled, Tailored Mobile Text Messaging to Reduce Problem Drinking (PD) is designed to develop and test a tailored adaptive text messaging/short message service (SMS) intervention for individuals interested in reducing their alcohol consumption.

Tailored interventions are individualized to the persons need states to make it more relevant and persuasive. Excessive alcohol consumption is a serious personal and public health issue and economic problem to society. Despite the significant consequences of problem drinking (PD), most persons with alcohol problems never seek formal treatment. While the emergence of internet based interventions (IBIs) has expanded access and brief intervention opportunities to PDs, they suffer from high attrition, limited ability to proactively meet individuals where PD occurs and adapt to real-time needs (e.g. high craving). Recent evidence from smoking cessation studies highlight that SMS interventions may provide the needed reinforcement to help individuals enhance self-regulation and reduce problem alcohol use. Moreover, there is evidence that since problem drinking has regular episodic patterns for which tailored interactive designs may be particularly effective.

OBJECTIVE(S)/SPECIFIC AIMS AND HYPOTHESES

This study is now in the recruitment phase. We are comparing different types of messaging in 240 PDs over a 12-week period. We are comparing 5 types of messaging: 1) tailored content and timed messaging adaptive to the participants current state (Beta Intervention); 2) Tailored content messaging (not adaptive or timed) sent at 6pm every day; 3) untailored consequence based messages sent at 6pm every day; 4) untailored benefit based messaging sent at 6pm every day and 5) Brief feedback and EMA only. In addition, we have a “goal only” group that does not mention alcohol. Web-based assessments will be completed at screening, baseline, at week 4, week 12 and through weekly EMA via SMS.

This is an exploratory study to generate effect sizes for a larger R01 trial. Our exploratory aims are as follows. Primary Aim: To test whether the Tailored-adaptive treatment arm is superior to EMA only and the content tailored only arm and the consequence and benefit messaging arms to EMA only in reducing weekly drinking/drinks per drinking day and percent days heavy drinking over the course of the 12 week intervention. Hypothesis 2a: Tailored-adaptive arm will be superior to the content tailored only and consequence and benefit messaging study arms in reducing weekly drinking/drinks per drinking day and heavy drinking days. Hypothesis 2b: Tailored-only messaging will be superior to consequence and benefit messaging arms in reducing weekly drinking/drinks per drinking day and heavy drinking days. Hypothesis 2c: All supportive messaging arms will be superior to assessment only in reducing weekly drinking/drinks per drinking day.

EXPERIMENTAL DESIGN

Overview: Participants are recruited through online recruitment on both social networking sites and alcohol screening sites. If a participant is interested they complete a brief survey online and if they are eligible they are instructed to call the researcher at a time that fits their schedule. The research assistant will review the

eligibility criteria and go over the consent form on the phone. Participants complete a 10 question quiz about the protocol in the form of a consent form quiz to ensure comprehension. They are also trained on filling out the weekly assessment text messages. Once enrolled and randomized, they receive messaging for 12 weeks and complete assessments at baseline, week 4 and week 12 as well as weekly EMA. Once individuals complete the study they will be given a $40 gift card to amazon. They are compensated $15 for the baseline survey, $$5 for the week 4 survey, and $15 for the week 12 survey. Primary outcomes include drinks per drinking day, days heavy drinking, and average drinks per week as measured through week EMA. Additional outcomes will include drinking related consequences, goal commitment and intervention satisfaction.

This study is now in the recruitment phase. We have already developed all messaging groups and done preliminary research on user preferences with 50 problem drinkers and 400 individuals on messaging preferences. This IRB protocol is only for the actual testing of the different forms of messaging in 240 individuals. The primary intervention group is the adaptive tailored group which includes messages tailored to a baseline survey results (including drinking times) and adaptive to ongoing drinking patterns and goal achievement via interactive ecological momentary assessment (EMA) which is real-time assessment and responses that are tailored to the persons current functioning. Other features include participant initiated help messaging and support network (e.g. spouse) alerts. We are testing the intervention with 240 PDs over a 12-week period. We are comparing 5 types of messaging: 1) tailored content and timed messaging adaptive to the participants current state (Beta Intervention); 2) Tailored content messaging (not adaptive or timed) sent at 6pm every day; 3) untailored consequence based messages sent at 6pm every day; 4) untailored benefit based messaging sent at 6pm every day and 5) Brief feedback and EMA only. In addition, we have a “goal only” group that does not mention alcohol. Web-based assessments will be completed at screening, baseline, at week 4, week 12 and through weekly EMA via SMS. All individuals are offered an additional 12 weeks of messaging in the group of their choice.

Description of Procedures:

Please note: This study is done completely over the web and phone. There are no office visits at all. It is all distant virtual interaction.

A brief bullet point review of general procedures is below:

- Recruit problem drinkers online
- Screen potential participants online
- Create phone screening appointment
- Offer consent form for review prior to call
- Screen participant on the telephone
- Obtain consent on the phone (participant completes after eligibility and it will be reviewed by staff prior to enrollment).

- Explain procedures (12 week study)
- Guide individual to complete baseline assessment
- Randomize participant
- Sign each individual participant up for the messaging program
- Review how to respond to assessment messages.
- Review procedures based on group assignment (What to Expect)
- Provide contact information of the RA & PI
- Email participants at week 4 to ensure safety and concerns (Week 4 Survey)
- Contact at week 12 for assessment (Week 12 Assessment)
- Offer referrals
- Offer additional 12 weeks of messaging without assessments.

Screening: Interested parties can learn about study recruitment through a number of web resources, including AlcoholScreening.org, Moderation Management, etc. Prospective participants will click on the ad to be sent to our secure web form, where they will be welcomed and given a brief description of the study aims and a warning not to complete the screening if intoxicated or in need of emergency care. If they choose to continue, they will be directed to generate a unique participant ID. They will be instructed to record this ID in a secure location, and proceed to the preliminary Screening Survey in Survey Monkey. If ineligible, they will be directed to a Survey Monkey page containing an extensive list of online resources and referrals. If eligible, they will be directed back into the Project AAIMS webform, where they will be required to provide their first name, initials, email and phone number. After doing so, they will be directed to a scheduling platform, where they can select a timeslot for an initial phone screening, to be completed with a member of the AAIMS staff. Upon selecting and confirming the appointment, they and the staff will be sent an email with the time and date of the phone appointment, and a reminder to read the Consent Form and complete the attached Consent Form Quiz prior to the call. The responsible AAIMS staff member will then call the prospective participant at the appointed time to go through the phone screening and determine eligibility. All forms with actual data are stored on the Survey Monkey server only with one's code. All other information is stored on a secure server with no questionnaire data.

Baseline Assessment: To complete the baseline assessment individuals login to survey monkey and enter their unique code to identify them. IP address is not collected. This will occur once the participant has confirmed their information with the research assistant who will provide them with the study website and code while they are on the phone. No codes will be given over email. The reason for this is to ensure participant information is confidential and secure. Once enrolled, participants will complete the baseline battery of measures described in the assessments section via the web-based assessment in their secure portal.

Similar to our current studies, the RAs will assess potential participants and when a question or concern is present, flag the case to have the clinician review the data and speak directly with the potential participant. RAs are trained to have a low threshold to speak with the clinician on duty and a clinician is available at all times during both

screening and follow-up appointments. We already had a double review of the first 20 clients where the RA and a trained therapist each independently spoke with the potential participant and there

Randomization: Once participants complete the baseline assessment they will receive the brief feedback on safe drinking levels and be automatically randomized into one of the five treatment arms stratified by gender, alcohol consumption, and readiness for change. The split for stratification is as follows: Gender: Male and Female; Severity: Above 24 drinks for men and above 21 drinks for women; Readiness score: 0-7 (low); 8-10 (high). Based on these scores separate envelopes are created (envelope randomization method), where the RA chooses an envelope and places the participant in one of the 5 study conditions randomly chosen. We stratify to ensure proper heterogeneity across groups. Each grouping has an equal number of study conditions to ensure any one group (e.g. male, high severity, high effort) is represented across groups. At the half-way point of the study, we will examine how well balanced each of the groups are with regard to the stratification variables and weight the conditions in the envelopes accordingly.

Individuals who drop out after randomization will not be replaced and we will conduct intent to treat analysis on all individuals who receive at least 1 day of text messages. While the research assistant will review the study arms with the participants in general during consent, individuals randomized to the different study arms will be given individualized instructions on what to expect. During the course of the intervention all participants will be allowed to call the RA for any reason and will be able to pause their messaging for 24 hour periods.

Follow-up Assessment: We will conduct a brief web-based week 4 assessment to assess drinking variables and overall functioning. This is 15-20 questions. Three days before their 12-week completion date participants will be sent a reminder text and email to complete the final assessment via a web-based survey. The week 12 assessment will include all the baseline measures as well as an item on perceived relevance of the intervention content to them specifically (Strecher, Shiffman, & West, 2006), treatment history during the intervention, overall recommendations to improve the intervention in addition to additional resources for PD. During the course of the study, additional data elements such as the number of eligible vs. ineligible participants, reasons for exclusion, referral sites, length of time to complete the screening and each assessment, and numbers of persons who drop out at any point in the study and at which point they drop out.

Ecological Assessment: Once the study begins all subjects will receive weekly ecological assessment for assessment only purposes (TA arm will also receive interactive assessment). Weekly Drinking: Three items will be sent every seven days including: The number of drinking days in the last week, the average number of drinks per drinking day in the last week, and number of drinking days where more than 5 drinks were consumed (4 for women) in the last week. We will also ask about

improvement over the course of the week to both assess change but also guide the adaptive intervention group. We chose this time to send messages during the day to reduce the chances that individuals will be intoxicated while reporting their drinking in their natural environment. Shiffman (2009) highlights the use of EMA to collect data from drinking and substance abusing populations and highlights while some have shown mild intervention effects (e.g. Collins et al., 1998) that most show little reactivity. Moreover, collecting weekly drinking assessments should reduce the likelihood of reactivity.

Study Treatments:

All SMS arms will first be given brief feedback on safe drinking levels in addition to messaging. This is specifically designed to test whether the addition of messaging enhances feedback on safe drinking but also ensure that all participants have access to a credible intervention. We will make every effort to reduce the chances that participants will be unblinded to their study condition – particularly individuals in the EMA condition. During the consent process we will inform participants that we are comparing different types of frequencies of text messaging including assessing drinking patterns and commitment through text messaging to see how it affects drinking.

Tailored Adaptive (TA): The TA treatment will be developed during the first phase of the study and tested in the RCT against the other study treatment arms. The intervention includes tailored messaging content and timing and frequency parameters initially based on a web-based assessment but adaptive based on current goal attainment and commitment. See Figure 1 in Human Subjects for a diagram of the intervention features. The messaging in the tailored adaptive will both be tailored to the baseline assessment results as well as to the participants current functioning. For example, a client low in confidence to change at baseline will receive messages to boost confidence. However, unlike in the TO condition, messages in the TA condition will change over the course of the study based on the participants current level of functioning. For example, if a participant responds to an SMS assessment message that they have a craving, they would receive craving messages (e.g. A craving does not mean you have to drink, it only means you have a craving) or if their confidence has increased that would NOT receive messages to boost their confidence in general.

Tailored Content Only (TO): The TO treatment arm will include tailored messaging based on the baseline assessment only sent at 6pm daily. Numerous IBIs and SMS studies have used baseline assessment information to tailor content throughout the intervention. Tailored content interventions are perceived as more relevant than untailored interventions. To our knowledge, this is the first study to compare a tailored adaptive over time intervention with multiple contacts to a baseline only limited contact tailored intervention. The messaging in the TO condition will be tailored to the baseline assessment results only. For example, a client low in confidence to change at baseline will receive messages to boost confidence throughout the study (e.g. Once you start to believe you can change, you can start the process of changing).

Consequence Messaging (CM): The consequence messaging arm will include messages on the consequences of PD sent at 6pm daily and is based on the previous literature using the Health Belief Model to induce change. Consequence based messaging was the experimental condition in the only messaging study (using PDAs) to date designed to reduce alcohol consumption. Moreover, in our own work we found that individuals who preferred consequence based messaging to benefit driven messaging scored lower on readiness for change which is consistent with the general print tailoring literature. Messages in this condition will highlight the consequences of alcohol use in various life domains (e.g. heavy alcohol use increases the risk of heart conditions OR heavy alcohol use is associated with poor sleep quality).

Benefit Messaging (CM): The benefits messages will be similar to the consequence messaging arm but will include messages on the benefits of changing sent at 6pm daily. In our own work we found that individuals who preferred benefit driven messaging scored higher on readiness for change which is consistent with the general print tailoring literature. Messages in this condition will highlight the benefits of decreasing of alcohol use in various life domains (e.g. When you control your drinking you end up waking up refreshed and ready for the day).

Ecological Momentary Assessment Only (EMA): The EMA only arm will include the four weekly EMA items (Commitment to change (1x week), drinking days, average drinks per drinking day, number of heavy drinking days). Because intensive EMA can be an intervention within itself due to ongoing self-monitoring EMA across all conditions will be limited to three days and five total messages including three messages about weekly drinking sent on Sunday early afternoon. The EMA condition is designed to test whether brief feedback and weekly monitoring can reduce PD compared to active SMS interventions. The EMA only condition will send individuals assessment messages only (e.g. how committed are you to not drink heavily in the next 24 hours or how many days this week did you drink alcohol).

No Alcohol Messages (Goal Only): Messages that are general goal directed messages without the mention of alcohol will be offered to all individuals who are concerned about confidentiality. At present - we have placed 15 people in this group. While we will assess this group compared to other groups, they will be analyzed separately because of forced randomization. The “Goal Only” group was created to protect the safety of participants who were concerned about confidentiality and includes messages that are very general and do not explicitly refer to drinking or alcohol e.g. “Remembering your goals tonight when you go out will help you realize them tomorrow”.

Measures & Schedule of Events (See Attached)

Measure	Construct	Time					
			Intervention Messaging Period				Optional Messaging
		Screen	Baseline (Randomize)	Weekly EMA	Week 4	Week 12	Weeks 12-24
Demographics	-	X	X			X	
QFV-30 questionnaire*	Alcohol consumptions last 30 days	X			X		
QFV-90 questionnaire*	Alcohol consumptions last 90 days		X			X	
EMA (Drinking Days, Drinks per Drinking Day, Heavy Drinking Days)	Weekly alcohol consumption			X			X
Frequency of Drug Use	Frequency drug use last 30 days	X				X	
Tx History & Medications	Substance abuse and mental health treatment history (last year) and current medications.	X				X	
Short Alcohol Withdrawal Scale*	Alcohol withdrawal potential	X					
Short Inventory of Problems*	Alcohol related consequences		X			X	
3-Item Primary Appraisal Measure: Alcohol	Subjective harm from use, continued use and benefit to stopping		X		X	X	
The Obsessive-Compulsive Drinking Scale*	Craving, salience and loss of control over alcohol		X			X	
Drinking Norms Rating Form	Perceptions of participants own and others drinking behavior		X			X	
Drinking Goals	Daily and weekly consumption goals	X	X		X	X	
Typical Drinking Patterns	When individuals are most likely to drink heavily		X		X	X	
Goal Commitment	Commitment to moderation and individual goals		X	X	X	X	
Single item rulers	Readiness to change, importance, and confidence to change.		X		X	X	
Triggers Questionnaire	Situations that may trigger heavy drinking		X			X	
Relevance of intervention to needs	Single item on whether material was relevant to participant				X	X	

**Other sources of help sought**	**Outside help sought during the intervention**					**X**	
**Satisfaction and recommendations**	**Overall program satisfaction and recommendations.**					**X**	

*Validated scales

A brief description of the site(s) where the research is to be conducted, including information about the adequacy of facilities for the safe and appropriate conduct of the research, and relevant demographic and epidemiological information about the country of region concerned.

INCLUSION AND EXCLUSION CRITERIA

Inclusion and exclusion criteria will be ascertained via: 1- a web-based screening form and 2- through a phone call with the research assistant.

Participants who respond to the internet advertisements or come directly to the study home page will complete a brief eligibility screener as well as a phone screening which will include QFV-30 questionnaire which assesses drinking patterns (Miller, 1996; 2 minutes) with examples of standard drinks, a face valid frequency of drug use scale within the last month, Short Alcohol Withdrawal Scale (Gossip et al., 2002; SAWS; score of 12 or above), and a brief assessment of mental health treatment and current medications. In addition, all potential participants will be asked about their messaging plan, if they are willing to receive between 15 to 120 text messages per month for the next three months (See Screening and Phone Screening Documents).

Inclusion: Participants must:
(a) be fluent and able to read in English at the eighth grade level; (Self report and consent form quiz)
(b) be between the ages of 21 and 65; (self-report; age in demographics)
(c) have an estimated average weekly consumption of greater than 12 or 15 standard drinks per week for women and men, respectively; (self-report; QFV-30 questionnaire)
(d) be willing to reduce their drinking to non-hazardous levels ; (Self-report)
(e) be willing to provide informed consent; and
(f) own a mobile phone and have an active email address and are willing to receive and respond to up to 120 text messages total per month (average about 50). (self-report in demographics questionnaire)

Exclusion: Participants will be excluded from the study if they:

(a) present with significant substance use or a current substance use disorder (for any substance other than alcohol, nicotine, or caffeine), which is defined as greater than once weekly use in the past month; (Frequency of Drug Use Questionnaire). Marijuana can be used up to 2x a week.

(b) present with a serious psychiatric illness or suicide risk as measured by previous inpatient treatment, medications for psychosis or recent suicidality; (Treatment history form & self-report; a current self-reported or clinician determined diagnosis of Major Depression or past or present bipolar disorder, delusional disorder or schizophrenia (self-report and clinical interview).

(c) demonstrate clinically severe alcoholism, as evidenced by physical withdrawal symptoms or a history of serious withdrawal symptoms (e.g., hallucinations, seizures, or delirium tremens), and score greater than 12 on the Short Alcohol Withdrawal Scale (SAWS).
(d) report a medical condition that precludes drinking any alcohol – including pregnancy (self-report) or
(e) Unable to understand research study procedures as evidenced a score of less than 7 out of 10 on the consent form quiz (consent form quiz).

RECRUITMENT PROCEDURES

Participants are recruited through online recruitment on both social networking sites and alcohol screening sites. If a participant is interested they create an anonymous unique code and complete a brief survey online and if they are eligible they are instructed to call the researcher at a time that fits their schedule.

INFORMED CONSENT

Electronic consent will be obtained after the phone call and the initial screening procedures and verbal consent will be obtained via the RA during the phone screening process. However, all participants will have a version of the consent to review prior to the phone call to ensure that they have the opportunity to understand the study in advance of the phone call. Electronic signatures or initials for consent are used in many types of legal, financial and healthcare settings. An electronic signature for the purpose of US law as "an electronic sound, symbol, or process, attached to or logically associated with a contract or other record and executed or adopted by a person with the intent to sign the record. For the purposes of this study, potential participants will both need to agree to the consent form, write in their initials, pass the consent form quiz, and give verbal assent to complete the consent process and be enrolled. If eligible based on the web and phone screening, participants will be given an online consent form to complete and sign.l. The research team will be responsible for obtaining from every participant prior to his/her participation in the study an informed consent signed by the participant with their initials AND assent on the phone , in accordance with the CFR, Title 45 Part 46. Consent will be obtained from the participant after a full explanation of the purpose of the study, risks and discomforts involved, potential benefits, etc. have been provided by the Investigator both verbally and in writing. Informed consent will be obtained by study staff on the phone. Once the

consent is completed, the RA will review the consent form quiz and call the participant to discuss any wrong answers or additional questions about the study.

DISCOMFORTS AND RISKS

There is some risk that participants will be identified as participants in a study of treatment for problem drinking via participant contact or through the mobile phone message or that the self-report assessments will adversely affect participants' well-being. Computerized assessment and messaging. The primary concern to confidentiality is that there may be an inadvertent breach of confidentiality concerning problem drinking behavior. It is possible that since participants will be receiving messages on their personal mobile phones, another person may view a message specific to changing alcohol use from an unguarded phone. While unlikely, it is possible that there may be a breach of confidentiality if a participant's home number is obtained through the computer database. Like all studies, individuals will be assigned to the assessment only group. However, they will receive guidelines on safe drinking. Rating scales and questionnaires Assessments are all non-invasive and add no special risk, although they do cover sensitive areas. The major disadvantages are the time taken to complete them, and possible breach of confidentiality. Our past experience indicates that these measures are acceptable to participants. Careful efforts to maintain confidentiality have been effective in our previous research and will be continued.

Participants will be recruited through the internet and before any messages are sent will be contacted by a research assistant to review the consent form – including an explanation of the study protocol, its risks, potential benefits, and alternative treatment. Additionally, all participants must complete the consent form quiz to ensure comprehension. An entire copy of the informed consent form will available for download by the participant. Protection against risk - Inclusion criteria and the use of trained research staff following the initial screening will minimize acceptance of participants with severe alcohol use disorders into the study. Participants with a history or current symptoms of alcohol withdrawal will be referred to more intensive care. During the intervention phase, participants' alcohol use will be monitored through EMA. If a participant reports drinking at greater level, the RA will call the participant. Participants will be given the emergency numbers of the PI and study RA for consultation. Participants who have dropped out of study treatment and are interested in returning to the program or attending another program will be referred for appropriate treatment according to their needs and wishes. We will not be asking individuals sensitive information about harm to self until we have contact information so we can contact them. Numerous studies have been conducted over the internet and mobile phones without any subject contact. There have been more internet based intervention studies for alcohol use than any other disorder. We have significantly

more information that most studies in that we will have actual contact with the individuals in the study by telephone prior to the study beginning then again at week 4 and week 12. Regular SMS reminders will be sent to individuals enrolled in all arms of the study to remind them 1) if they want standard treatment, they can call the RA and be helped with referrals and 2) if they are worsening clinically, they can call for help. If a participant is threatening to harm themselves or another we will use standard protocol to inform the proper authorities as if it was a patient through phone contact as we will have their address and phone number. This will be determined based on email, phone, mobile or web-based data from the participant. We have had no adverse events with the first 104 individuals enrolled.

POTENTIAL BENEFITS

Benefits to participants include additional monitoring and reminders to help them reduce their drinking. Based on the efficacy of previous messaging interventions for smoking cessation, it is possible that participants reduce the personal and public consequences associated with problem drinking. We expect that the brief feedback and EMA only group may also find benefit in the extra monitoring. Moreover, all participants have their choice of messages for 12 weeks after the study period. If the intervention is found to be useful to participants, it will provide a minimal resource automated intervention for those seeking to reduce their drinking.

DISCONTINUATION OF STUDY/SUBJECT WITHDRAWAL

If a participant self-reports worsening of their condition as a result of the study procedures they will be withdrawn. All participants data will be reviewed at week 4. For those who report their condition has gotten worse, we will call them and inquire about their current symptoms and discontinue them from the study if necessary. Participants can withdraw at any time and we provide referrals. We provide referrals to internet screening sites and treatment using the SAMHSA Treatment finder. We inquire about reasons for withdrawing.

ADVERSE EVENTS

The Principal Investigator and Co-Investigators are clinical psychologists and licensed Ph.D. social workers and will be responsible for distinguishing between a Serious Adverse Event (SAE) and a non-serious adverse event (AE). All SAE and AE will be reported to the IRB of the NSLIJ according to NSLIJ HRPP policies and to the National Institute of Alcohol Abuse and Alcoholism (NIAAA) project officer within 48 hours of its occurrence. Additionally, the PI will prepare an annual report summarizing all SAE and AE to be submitted to the NIAAA project officer and the IRB. In addition to preparing for SAE, participants' alcohol use will be monitored. Participants will be referred for appropriate treatment according to their needs and wishes if they show severe deterioration, and continued evaluation of participants through assessments will provide ongoing information concerning their clinical status. Significant deterioration is

defined as a significant increase in drinking since the baseline interview; or other indicators through any interaction with the research staff. If any of these indicators are present, the participants will be immediately withdrawn from the study and referred to immediate and appropriate treatment. Primary responsibility and decision making in regards to participants remaining in or being withdrawn from the study will lie with the principal investigator.

DATA SAFETY MONITORING

The protocol has been reviewed by study staff, external consultants and the IRB. In addition, an independent Data and Safety Monitoring Board (DSMB) has been formed. No members are otherwise affiliated with the study. Each of the DSMB members have had extensive clinical and/or research experience with the study population. The members of the board include the following:

- Milton Wainberg, M.D., Assistant Clinical Professor of Psychiatry, Columbia University College of Physicians and Surgeons.
- Aaron Hogue, Ph.D., Associate Director of Health Services Research, National Center on Addiction and Substance Abuse at Columbia University.

The DSMB shall determine safe and effective conduct and recommend conclusion of a trial if significant risks develop or the trial is unlikely to be concluded successfully. Specifically, the data and safety monitoring board and PI shall be responsible for the following:

- Data monitoring that will take place on a regular basis (every 6 months after recruitment or the halfway point of the trial).
- Evaluating the progress of the trial, participant risk versus benefit, and other factors that may affect study outcome. Monitoring may also consider factors external to the study when interpreting the data, such as scientific or therapeutic developments that may have an impact on the safety of the participants or ethical issues related to the study.
- Inquiring for further information as necessary to accomplish their mission.
- Maintaining confidentiality during all phases of the trial including the monitoring, preparation of interim results, review and response to monitoring recommendations.

CRITERIA FOR EVALUATING RESPONSE/STATISTICAL ANALYSIS

The aims of this proposal are consistent with the initial sub-stages of Stage I developmental research described in the Stage Model of Behavioral Research (Rounsaville, Carroll & Onken, 2001). <u>Primary Aim 1:</u> To develop an adaptive text messaging program for problem drinking. 1A. Develop a brief assessment, normative feedback and list of text messages based on the PD intervention literature & obtain outside review from thought leaders. 1B. Survey 40 problem drinkers on overall SMS intervention preferences including message content and timing and frequency

preferences. 1C. Develop SMS intervention, review with thought leaders and pre-pilot and test with 10 problem drinkers and modify intervention based on feedback. Primary Aim 2: : To test whether the Tailored-adaptive treatment arm is superior to the content tailored only arm and the consequence messaging arm in reducing weekly drinking/drinks per drinking day and percent days heavy drinking over the course of the 12 week intervention. Hypothesis 2a: Tailored-adaptive (TA) arm will be superior to the content tailored only and consequence messaging study arms in weekly drinking/drinks per drinking day. Hypothesis 2b: Tailored-only messaging will be superior to consequence messaging in reducing weekly drinking/drinks per drinking day. Hypothesis 2c: All supportive messaging arms will be superior to assessment only in reducing weekly drinks weekly drinking/drinks per drinking day. Hypothesis 2a: TA will be superior to TO & CM in reducing weekly drinking/drinks per drinking day. Hypothesis 2b: TO will be superior to CM in reducing weekly drinks. Hypothesis 2c: All supportive messaging arms will be superior to EMA only in reducing weekly drinking/drinks per drinking day. Other outcomes include percent heavy drinking days and drinking days.

Data will be cleaned, summary variables created, missing data evaluated, and distributions assessed for normality. Equivalence of the random assignment of groups on key baseline characteristics will be assessed, and in the unlikely event that groups differ significantly on any characteristics, we will run analyses both with and without these variables as covariates to determine whether baseline differences may account for differences in outcomes. Gender and race/ethnicity differences will be evaluated and these variables included as covariates if they are correlated with outcomes. We will aggregate daily EMA into use weekly summary data, as it allows us to examine the trajectory of drinking patterns week by week over the course of the 12-week study.

Data Management & Analysis

Analyses will be conducted separately on two overlapping samples. All randomized subjects who received at least two days of text messages will be included in the first set of analyses as long as they have some follow-up data on primary analyses. This represents our main outcome analysis. We also will conduct a “worst-case” analysis in which subjects lost to attrition will use their pre-treatment drinking as their week 12 drinking data. Finally, in step three, we will conduct “completer analyses” on those patients completing a full course of messaging. This latter approach to analysis, although subject to more bias, attempts to provide an estimate of the maximal effect of the treatments under ideal conditions. Similar results with all three approaches would increase confidence in the findings. Effect size estimates along with significance levels will be generated for all comparisons. A goal of the study is to determine a reasonable effect size estimate and to assist in planning larger studies of the intervention if findings justify that next step. The critical effect size estimate is of drinking outcomes. The sample sizes of 30-40 per group will provide reasonable estimates of the differences in means.

There are two primary outcome variables: 1) Average drinks per week (sum of drinks drinking day) over the course of the study and 2) the percent of total days in which there is heavy drinking over the course of the study; This data will be collected via the Baseline, Week 4 and Week 12 assessments. Various summary statistics will also be computed, and significantly skewed distributions will be appropriately transformed prior to statistical analysis (e.g. Shapiro–Wilk test). As exploratory secondary analyses we will compare study arms on differences in key secondary outcome variables assessed at baseline and week 12 including alcohol related consequences and drinking norms.

Two sets of preliminary analyses will be performed. The first will determine whether there are important differences among the experimental cells despite randomization. Pretreatment drinks per week, heavy drinking days, effort to reduce drinking and demographic characteristics (age, gender) will be examined using two-way analyses of variance in order to measure comparability among the five groups (and the goal only group) and success of the randomization procedure. Data will also be examined for conformity to statistical assumptions of normality, and a square root transformation will be performed on skewed data. To further account for skewness, zero-inflation, or other departures from Normal response distributions, we will use response transformations or more complex models.

This study is exploratory and meant to generate effect sizes. However, our primary exploratory hypothesis is that there will be an ordered effect across conditions such that TA results in a significantly greater reductions in drinking than TO, TO greater than CM, and CM greater than EMA only. The GO group will be analyzed separately and compared to each condition using logistical regression and entered into the full model with all conditions as a secondary analysis. Effect size estimates along with significance levels will be generated for all comparisons. A goal of the study is to determine a reasonable effect size estimate, to assist in planning larger studies of the intervention, if findings justify that next step. The critical effect size estimate is of drinking outcomes. The sample sizes of 30-40 per group (after 20% attrition) will provide reasonable estimates of the differences in means. We will carefully examine the issue of missing data using a random-effects approach to modeling different patterns of missed data across the data collection points of the study as specified by Hedeker and Gibbons (1997). As exploratory secondary analyses we will compare study arms on differences in key secondary outcome variables assessed at baseline and week 12 including week 12 alcohol related consequences and drinking norms among others using multiple linear regression with baseline values and control variables entered into the first step and dummy coded treatment arms entered into the second step. Because this is an intervention development study, we will also compare groups at week 12 on overall satisfaction with the intervention and perceived relevance of the intervention to their needs using ANCOVA.

CASE REPORT FORMS

See attached for all forms

CONFIDENTIALITY

There have been multiple studies using SMS as a means to communicate patient information of much more sensitive topics (e.g. http://clinicaltrials.gov/ct2/show/NCT01229722) and numerous studies where messages relate to specific disease topics such as diabetes (Ferrer-Roca et al.2004; Franklin et al. 2007; Hanauer, Wentzell, Laffel & Laffel, 2009; Kim & Jeong, 2007; Kwon et al., 2004), asthma (Anhoj & Moldrup 2004; Ostojic, Cvoriscec, Ostojic, Reznikoff, Stipic-Markovic et al., 2005; Ryan et al., 2005), obesity and weight loss (Joo & Kim 2007; Patrick et al., 2009), tobacco dependence (Knight, Free et al., 2011; Obermayer et al., 2004; Riley, Obermayer & Jean Mary, 2008; Rodgers et al. 2005). In all of these studies, SMS reminders were sent through the traditional telephone company networks as part of the research study. Individuals who are concerned about the use of the word alcohol within text messages will be offered the ability to send receive mirrored messages without the mention of alcohol. Currently, about 20% of participants are choosing this option. Participants will have the option to NOT mention drinking alcohol in the messages or use codes like “how many cups of coffee did you have last night?” as a way to ask how many drinks they had. Messages about change will be general – e.g. “think about how changing will improve your energy levels”. However, we will follow confidentiality practices of messaging studies on sensitive topics. Traditional text messages may be viewed by a third party if seen on an individual’s phone. To reduce any possible breaches of confidentiality, participants will be offered instructions on how to :1. Change message settings to alert individuals that an SMS message has been received but do NOT display or preview any of the message. This ensures that a third party cannot passively see the message on a participants phone because no information is provided. 2. Inform individuals to add a security code to their phone in which one must enter the security code to view a message. 3. Inform individuals to create a contact to which they would feel comfortable receiving a messages. For example, they can program their phone to display the name, Bob Jones every time we send them a message. 4. Inform the participants that they should keep their phone on their person or in a secure location at all times. 5. Inform participants on how to delete all messages from a particular sender.

Separate databases are used for code creation, survey completion, and text messages. All electronic files (e.g., database, spreadsheet, etc.) containing identifiable participant information are password protected. All computers hosting such files have a BIOS password to prevent access by unauthorized users. Furthermore, for systems not running Windows 2000/XP, a password-protected screen saver will be installed

and configured to activate ten minutes after the computer has been idle. Research staff will have a secure login to access study data. Participant data via the web-based assessment will be stored on secure servers. The database for text messaging uses an Internet Information Server (IIS) structure to authenticate and assign the proper groupings to the logins. At no point does the program ask clients for their name or contact information, other than phone number and email. The database server for SMS is hosted in a data center secured using biometric methods and compartmentalized cages. The server is protected from spyware, malware and viruses using XXXX from TrendMicro. Rip Road follows best practices and industry-standard methods for fault-tolerance and scalability as well as software application design and implementation. Data is stored in a Microsoft SQL Server database and is secured using SQL Authentication. There is an advanced Router system in place that tracks (via solar winds) both intrusion attempts to guard against unauthorized attempts to access data. There are nightly database backups to a remote server protect against data loss. All electronic research files (e.g., database, spreadsheet, etc.) containing identifiable participant information are password protected. All computers hosting such files have a password to prevent access by unauthorized users. When participant data are exchanged with others, the data will be coded. All data if transported will be encrypted while en-route to the recipient with strong encryption levels (≥ 128 bits for symmetric encryption (DES) and ≥ 1024 bits for asymmetric encryption (RSA)). Participants will be informed of alternatives to participation and given appropriate resources. A certificate of confidentiality has been obtained for this study (expires 5/2019).

DATA DISCLOSURE/PUBLICATION

PHI will not be disclosed during publication and all data will be identified. Identifiable PHI is only collected by our SMS provider RIPROAD.

CONFLICT OF INTEREST

See COI form. Dr. Muench consults with mobile health companies and recently received his company back after selling it. He has no conflict of interest with the company hired to provide the messaging services. He has completed a COI plan.

REFERENCES/BIBIOGRAPHY

Abraham, C. & Michie, S. (2008). A taxonomy of behavior change techniques used in interventions. H*ealth Psychology, 27*, 379-387.

Adler, R. (2009). The Landscape of Texting for Health, In Fogg, B. J., & Adler, R. (Eds.). *Texting 4 Health*. Stanford, California: Stanford Captology Media.

Ajzen, I. (1991). The theory of planned behavior. *Organizational Behavior and Human Decision Processes, 50*, 179–221.

Anhoj, J. & Moldrup, C. (2004). Feasibility of collecting diary data from asthma patients through mobile phones and SMS (short message service): response rate analysis and focus group evaluation from a pilot study. *Journal of Medical Internet Research, 6*(4), e42.

Anton, R. F. (2000). Obsessive-compulsive aspects of craving: Development of the Obsessive Compulsive Drinking Scale. *Addiction, 95*(Supp. 2), S211-S217.

Badami, R., VaezMousavi, M., Namazizadeh, M., & Wulf (in press). Feedback after good versus poor trials: Differential effects on self-confidence and activation. *Research Quarterly for Exercise and Sport*.

Badami, R., VaezMousavi, M., Wulf, G., & Namazizadeh, M. (in press). Feedback after good trials enhances intrinsic motivation. *Research Quarterly for Exercise and Sport.*

Baer, J. S., Stacy, A., & Larimer, M. (1991). Biases in the perception of drinking norms among college students. *J Stud Alcohol, 52,* 580–586.

Bandura A. Self-efficacy: The exercise of control. New York: W H Freeman/Times Books/Henry Holt & Co; 1997.

Bartholomew, L. K., Parcel, G. S., Kok, G., & Gottlieb, N. H. (2006). Pla*nning health promotion programs: An intervention mapping approach.* San Francisco, CA: Jossey-Bass.

Bates, M. E., Bowden, S. C., & Barry, D. (2002). Neurocognitive impairment associated with alcohol use disorders: implications for treatment. *Experimental and Clinical Psychopharmacology, 10*(3), 193-212.

Bauer, S., de Niet, J., Timman, R., & Kordy, H. (2010). Enhancement of care through self-monitoring and tailored feedback via text messaging and their use in the treatment of childhood overweight. *Patient Education and Counseling, 79*(3), 315-319.

Baumeister, R. F., & Vohs, K. D. (Eds.). (2004). *Handbook of selfregulation. Research, theory, and applications*. New York: The Guilford Press.

Berkman, E. T., Dickenson, J., Falk, E. B., & Lieberman, M. D. (2011). Using SMS text messaging to assess moderators of smoking reduction: Validating a new tool for ecological measurement of health behaviors*. Health Psychology, 30*(2), 186–194.

Bernstein E., Bernstein, J. & Levenson, S. (1997). Project ASSERT: An ED-based intervention to increase access to primary care, preventive services, and the substance abuse treatment system. *Annals of Emergency Medicine, 30*(2), 181-197.

Bewick, B. M., Trusler, K., Barkham, M., Hill, A. J., Cahill, J., & Mulhern, B. (2008). The effectiveness of web-based interventions designed to decrease alcohol consumption-a systematic review. *Prev Med, 47*(1), 17-26.

Bickmore, T., Mauer, D., Crespo, F., & Brown, T. (2007). Persuasion, Task Interruption and Health Regimen Adherence. *Lecture Notes in Computer Science*: *Persuasive Technology*, 1-11.

Brendryen, H., Kraft, P., & Schaalma, H. (2010). Looking inside the black box: Using intervention mapping to describe the development of an automated smoking cessation intervention happy ending. *The Journal of Smoking Cessation, 5*, 29–56.

Brown, S. A., Christiansen, B. A., Goldman, M. S. (1987). The Alcohol Expectancy Questionnaire: An instrument for the assessment of adolescent and adult alcohol expectancies. *Journal of Studies on Alcohol, 48*(5), 483-491.

Burton, C., Weller, D., & Sharpe, M. (2007). Are electronic diaries useful for symptom research? A systematic review*. J Psychosom Res, 62*, 553–561.

Büssing, A., Girke, M., Heckmann, C., Schad, F., Ostermann, T., & Kröz, M. (2009). Validation of the self regulation questionnaire as a measure of health in quality of life research. *European Journal of Medical Research, 14*(5), 223-227.

Carey, K. B., Scott-Sheldon, L. A., Elliott, J. C., Bolles, J. R., & Carey, M. P. (2009). Computer-delivered interventions to reduce college student drinking: a meta-analysis. *Addiction.* [Epub ahead of print].

Carver, C. S., & Scheier, M. F. (1998). *On the self-regulation of behavior*. Cambridge, UK: Cambridge University Press.

Cole-Lewis, H. & Kershaw, T. (2010). Text messaging as a tool for behavior change in disease prevention and management. *Epidemiologic Reviews, 31*(1), 59-69.

Collins, R. L., & Muraven, M. (2007). Ecological momentary assessment of alcohol consumption. In A. A. Stone, S. Shiffman, A. A. Atienza, & L. Nebling (Eds.) *The science of real-time data capture: Self reports in health research* (189-203). New York: Oxford University Press.

Colzato, L. S., van den Wildenberg, W., & Hommel, B. (2007). Impaired inhibitory control in recreational cocaine users. *PLoS ONE,* http://www.plosone.org/doi/pone.0001143

Cunningham, J. A., & Breslin, F. C. (2004). Only one in three people with alcohol abuse or dependence ever seek treatment. *Addictive Behaviors, 29*(1).

Cunningham, J. A., Humphreys, K., Kypri, K., & van Mierlo, T. (2006). Formative evaluation and three-month follow-up of an online personalized assessment feedback intervention for problem drinkers*. J Med Internet Res, 8*(2), e5.

Cunningham, J., Wild, T., Cordingley, J., van Mierlo, T., & Humphreys, K. (2009). A randomized controlled trial of an internet-based intervention for alcohol abusers. *Addiction, 104*(12), 2023-2032.

Danaher, B. G., & Seeley, J. R. (2009). Methodological issues in research on web-based behavioral interventions. *Ann Behav Med., 38*(1), 28-39.

Dawson, D. A., Grant, B. F., Stinson, F. S., & Chou, P. S. (2005). Psychopathology associated with drinking and alcohol use disorders in the college and general adult populations. *Drug Alcohol Dependence, 77*(2), 139-150.

DiClemente, C. C. (1991). Motivational interviewing and the stages of change. In W.R. Miller & S. Rollnick (Eds.), *Motivational interviewing: Preparing people to change addictive behavior* (pp. 191–202). New York: Guilford Press.

Elliott, J. C., Carey, K. B., Bolles, J. R. (2008). Computer-based interventions for college drinking: A qualitative review. *Addictive Behaviors, 33,* 994–1005.

Everitt, B. J., & Robbins, T. W. (2005). Neural systems of reinforcement for drug addiction: from actions to habits to compulsion*. Nat. Neurosci. 8,* 1481–1489.

Ferrer-Roca, O., Cardenas, A., Diaz-Cardama, A., & Pulido, P. (2004). Mobile phone text messaging in the management of diabetes. *J Telemed Telecare, 10,* 282–285.

Fjeldsoe, B. S., Marshall, A. L., & Miller, Y. D. (2009). Behavior change interventions delivered by mobile telephone short-message service. *American Journal of Preventive Medicine, 36*, 165-173.

Franken, Hendrisksa, & van den Brink (2002). Initial validation of two opiate craving questionnaires the obsessive compulsive drug use scale and the desires for drug questionnaire. *Addictive Behaviors, 27*(5), 675-685.

Franklin, V. L., Waller, A., Pagliari, C., & Greene, S. A. (2007). A randomized controlled trial of Sweet Talk, a text-messaging system to support young people with diabetes. *Diabetic Medicine, 23*, 1332-1338.

Free, C., Knight, R., Robertson, S., Whittaker, R., Edwards, P., Zhou, W., Rodgers, A., Cairns, J., Kenward, M. G., & Roberts, I. (2011). Smoking cessation support delivered via mobile phone text messaging (txt2stop): A single-blind, randomized trial. *Lancet, 378*(9785), 49-55.

Freedman, M. J., Lester, K. M., McNamara, C. Milby, J. B., & Schumacher, J. E. (2006). Cell phones for ecological momentary assessment with cocaine-addicted homeless patients in treatment. *Journal of Substance Abuse Treatment, 30*, 105-111.

Fry, J. P., & Neff, R.A. (2009). Periodic prompts and reminders in health promotion and health behavior interventions: a systematic review*. J Med Internet Res., 11*(2), e16.

Furukawa, T. A., Kessler, R. C., Slade, T., & Andrews, G. (2003). The performance of the K6 and K10 screening scales for psychological distress in the Australian National Survey of Mental Health and Well-Being. *Psychol Med, 33*, 357–362.

Gold, J., Lim, M., Hocking, J., Keogh, L., Spelman, T., & Hellard, M. (2010). Determining the impact of text messaging for sexual health promotion to young people sexually transmitted diseases.

Gossop, M., Keaney, F., Stewart, D., et al (2002). A Short Alcohol Withdrawal Scale (SAWS): Development and psychometric properties*. Addiction Biology, 7*, 37-43.

Gross, J. J., & John, O. (2003). Individual differences in two emotion regulation processes: Implications for affect, relationships, and well-being. *Journal of Personality and Social Psychology, 85*, 348–362.

Haller, D. M., Sanci, L.A., Patton, G. C., & Sawyer, S. M. (2009). Text message communication in primary care research: A randomized controlled trial. *Family Practice, 26*, 325-330.

Hammersley R. (1994). A digest of memory phenomena for addiction research. *Addiction, 89*, 283–293

Heather, N., Smailes, D., & Cassidy, P. (2008). Development of a Readiness Ruler for use with alcohol brief interventions*. Drug and Alcohol Dependence, 98*(3), 235-240.

Heron, K. E., & Smyth, J.M. (2010). Ecological momentary interventions: Incorporating mobile technology into psychosocial and health behaviour treatments. *Br J Health Psychology, 15*, 1-39.

Hester, R. K., Squires, D. D., & Delaney, H. D. (2005). The computer-based drinker's check-up: 12-month outcomes of a clinical trial with problem drinkers. *Journal of Substance Abuse Treatment, 28*(2), 159-169.

Hill, T., Lewicki, P., Czyzewska, M., & Boss, A. (1989). Self-perpetuating development of encoding biases in person perception. *Journal of Personality and Social Psychology, 57*, 373–387

Imel, Z. E., Wampold, B., Millde, S. D., & Fleming, R. R. (2008). Distinctions without a difference: Direct comparisons of psychotherapies for alcohol use disorders. *Psychology of Addictive Behaviors, 22*(4), 533–543.

Koshy, E., Car, J., & Majeed, A. (2008). Effectiveness of mobile-phone short message service (SMS) reminders for ophthalmology outpatient appointments: Observational study. *BMC Ophthalmology. 8,* 9.

Kraft, P., Drozd, F., & Olsen, E. (2009). ePsychology: Designing theory based health promotion interventions. *Communications of the Association for Information Systems, 24*, 399–426.

Krebs, P., Prochaska, J. O., & Rossi, J. S. (2010). A meta-analysis of computer-tailored interventions for health behavior change*. Prev Med., 51*(3-4), 214-221.

Kreuter, M. W., & Wray, R. J. (2003). Tailored and targeted health communication: Strategies for enhancing information relevance. *Am J Health Behav, 27,* S227-S232.

Krishna, S., Boren, S. A., & Balas, E. A. (2009). Healthcare via cell phones: A systematic review. *Telemedicine Journal & E-Health, 15*, 231-240.

Latimer, A. E., Brawley, L. R., & Bassett, R. L. (2010). A systematic review of three approaches for constructing physical activity messages: What messages work and what improvements are needed? *Int J Behav Nutr Phys Act, 7,* 36.

Leong, K. C., Chen, W. S., Leong, K. W., Mastura, I., Mimi, O., Sheikh, M. A., Zailinawati, A. H., Ng, C. J., Phua, K. L., & Teng C. L. (2005). The use of text messaging to improve attendance in primary care: a randomized controlled trial. *Family Practice, 23,* 699–705.

Liang, H., Xue, X., & Berger, B. (2005). Web-based intervention support system for health promotion. *Decision Support Systems, 42*(1), 435-449.

Linke, S., Murray, E., Butler, C., & Wallace, P. (2007) Internet-based interactive health intervention for the promotion of sensible drinking: patterns of use and potential impact on members of the general public. *J Med Internet Res, 9*(2), e10.

Marlatt, G.A. (1985). Relapse prevention: Theoretical rationale and overview of the model. In G.A. Marlatt, & J.R. Gordon (Eds.), Relapse prevention (pp. 3–70). New York: Guilford.

Martindale, C. (1991). Cognitive psychology; a neural network approach. Pacific Grove CA: Brooks/Cole Publishing.

McKay, J. R. (2009). *Adaptive continuing care and the management of substance use disorders*. American Psychological Association Press. Washington DC.

McKay, J. R. (2009). Continuing care research: What we've learned and where we're going. *Journal of Substance Abuse Treatment, 36*, 131-145.

Michie, S., & Abraham, C. (2008). Advancing the science of behaviour change: A plea for scientific reporting. *Addiction, 103*, 1409–1410.

Miller, W. R., & Del Boca, F. K. (1994). Measurement of drinking behavior using the Form 90 family of instruments. *J Stud Alcohol, 12,* 112–118.

Miller, W.R., Tonigan, J.S., & Longabaugh, R. (1995). The Drinker Inventory of Consequences (DrInC): An instrument for assessing adverse consequences of alcohol abuse. Test manual. Rockville, MD: NIAAA Project MATCH Monograph Series Volume 4.

Moos, (2007). Theory-Based Processes that Promote the Remission of Substance Use Disorders. *Clin Psychol Rev*., 27(5): 537–551.

Moos, R. H., & Moos, B. S. (2006). Treated and untreated individuals with alcohol use disorders: Rates and predictors of remission and relapse. *International Journal of Clinical and Health Psychology, 6*(3), 513-526.

Morgenstern, J., & McKay, J. R. (2007). Rethinking the paradigms that inform behavioral treatment research for substance use disorders. *Addiction, 102,* 1377–1389.

Moss, H. B., Chen, C. M., & Yi, H. (2007). Subtypes of alcohol dependence in a nationally representative sample. *Drug and Alcohol Dependence, 91*(2-3), 149-158.

Murphy, Lynch, McKay, Oslin, TenHave (2007). Developing Adaptive Treatment Strategies in Substance Abuse Research, *Drug and Alcohol Dependence, 88*(2), S24-S30.

Neighbors, C., Lewis, M. A., Bergstrom, R. L., & Larimer, M. E. (2006). Being controlled by normative influences: Self-determination as a moderator of a normative feedback alcohol intervention. *Health Psychology, 25*, 571−579.

Nielsen (2008). *Nielsen Mobile Survey*. Retrieved from http://www.nielsenmobile.com/html/insights.html.

Noar, S. M., Benac, C. N., & Harris, M. S. (2007). Does tailoring matter? Meta-analytic review of tailored print health behavior change interventions. *Psychol Bull, 133*, 673-693.

O'Keefe, D. J. (2011). Generalizing about the persuasive effects of message variations: The case of gain-framed and loss-framed appeals. In T. van Haaften, H. Jansen, J. de Jong, & W. Koetsenruijter (Eds.), *Bending opinion:*

Essays on persuasion in the public domain (pp. 117-131). Leiden, The Netherlands: Leiden University Press

Patrick, K., Griswold, W. G., Raab, F., & Intille, S. S. (2008). Health and the mobile phone. *American Journal of Preventative Medicine*, 35(2), 177-181.

Patrick, K., Raab, F., & Adams, M.A. (2009). A text message-based intervention for eight loss: Randomized controlled trial. *Journal of Medical Internet Research, 11*, e1.

Pew Internet & American Life Project (2010). http://www.pewInternet.org/~/media//Files/Reports/2010/PIP_Mobile_Access_2010.pdf.

Pinjnenborg, G. H. M., Withaar, F. K., Brouwer, W. H., Timmerman, M. E., van den Bosch, R. J., & Evans, J.J. (2010). The efficacy of SMS text messages to compensate for the effects of cognitive impairments in schizophrenia. *British Journal of Clinical Psychology, 49*, 259-272.

Portnoy, D., Scott-Sheldon, L., Johnson, B. & Carey, M. (2008). Computer-delivered interventions for health promotion and behavioral risk reduction: A meta-analysis of 75 randomized controlled trials, 1988--2007. *Preventive Medicine, 47*(1), 3-16.

Prestwich, A., Perugini, M., & Hurling, R. (2009). Can the effects of implementation intentions on exercise be enhanced using text messages? *Psychology and Health, 6*, 677-687.

Prestwich, A., Perugini, M., & Hurling, R. (2010). Can implementation intentions and text messages promote brisk walking? A randomized trial. *Health Psychology, 29*, 40-49.

Prochaska, J. O., & DiClemente, C. C. (1983). Stages and Processes of Self-Change of Smoking: Toward an Integrative Model of Change. *Journal of Consulting and Clinical Psychology, 51*, 390-395.

Prochaska, J. O., DiClemente C. C., Velicer.W. F., & Fava J. (1988). Measuring processes of change: Applications to the cessation of smoking. *Journal of Consulting and Clinical Psychology, 56*, 520-528.

Riley, W. T., Rivera, D. E., Atienza, A. A., Nilsen, W., Allison, S. M., & Mermelstein, R. (2011). Health behavior models in the age of mobile interventions: are our theories up to the task? *Transl Behav Med.,1*(1), 53-71.

Riper, H., Spek, V., Boon, B., Conijn, B., Kramer, J., Martin-Abello, K., & Smit, F. (2011). Effectiveness of E-self-help interventions for curbing adult problem drinking: a meta-analysis. *J Med Internet Res., 13*(2), e42.

Riper, H., van Straten, A., Keuken, M., Smit, F., Schippers, G., & Cuijpers, P. (2009). Curbing problem drinking with personalized-feedback interventions: A meta-analysis. *Am J Prev Med, 36*(3), 247-255.

Robinson, S., Perkins, S., Bauer, S., Hammond, N., Treasure, J. & Schmidt, U. (2006). Aftercare intervention through text messaging in the treatment of bulimia

nervosa - Feasibility Pilot. *International Journal of Eating Disorders, 39,* 633-638*.*

Rodgers, A., Corbett, T., Bramley, D., Riddell, T., Wills, M., Lin, R., & Jones., M. (2005). Do u smoke after txt? Results of a randomized trial of smoking cessation using mobile phone text messaging. *Tobacco Control, 14*, 255-261.

Rooke, S., Thorsteinsson, E., Karpin, A., Copeland, J., & Allsop, D. (2010). Computer-delivered interventions for alcohol and tobacco use: a meta-analysis. *Addiction, 105*(8), 1381-1390.

Room, R., Babor, T., & Jürgen, R. (2005). Alcohol and public health. *The Lancet, 365*(9458), 519-530. doi: 10.1016/S0140-6736(05)17870-2

Rothman, A. J., Baldwin, A. S., & Hertel, A. W. (2004). Self-regulation and behavior change. Disentangling behavioral initiation and behavioral maintenance. In R.F. Baumeister, & K.D. Vohs (Eds.), *Handbook of self-regulation: Research, theory, and applications* (pp. 130–48). New York: Guilford Press.

Rounsaville, B. J., Carroll, K. M., & Onken, L. S. (2001). A stage model of behavioral therapies research: Getting started and moving on from stage I. *Clinical Psychology: Science and Practice, 8*(2),133-142.

SAMHSA, Substance Abuse and Mental Health Services Administration, *Results from the 2010 National Survey on Drug Use and Health: Summary of National Findings*, NSDUH Series H-41, HHS Publication No. (SMA) 11-4658. Rockville, MD: Substance Abuse and Mental Health Services Administration, 2011.

Schwartz, J. E., & Stone, A. A. (2007). Analysis of real-time momentary data: A practical guide. In A.A. Stone, S. Shiffman, Atienza, A. & Nebeling, L. (Eds.) *The science of real-time data capture*. Oxford University Press.

Shapiro, J. R. & Bauer, S. (2010). Use of SMS-based interventions to enhance CBT. In J. Bennett, H. Christensen, P. Farrand, K. Griffiths, D. Kavanagh, B. Klein, M. Lau, J. Proudfoot, D. Richards, J. White & C. Williams (Eds.), Oxford Guide to Low Intensity CBT Interventions. Oxford University Press.

Shiffman, S., Stone, A. A., & Hufford, M. R. (2008). Ecological momentary assessment. *Annual Rev Clin Psychol, 4,* 1-32.

Slattery, J., Chick, J., Cochrane, M., Craig, J., Godfrey, C., Kohli, H., et al. (2003). Prevention of Relapse in Alcohol Dependence. *Health Technology Assessment, Report 3*. Glasgow: Health Tech. Board for Scotland.

Sobell, M. B., & Sobell, L.C. (1993). Problem Drinkers: Guided Self-Change Treatment. New York: Guilford Press.

Sobell, L. C., and Sobell, M. B, eds. Alcohol consumption measures. In: Allen, J.P., and Columbus, M., eds. Assessing Alcohol Problems: A Guide for Clinicians and Researchers. Treatment Handbook Series 4. Bethesda, MD: National Institute on Alcohol Abuse and Alcoholism, 1995.

Stacy, A. W. (1997). Memory activation and expectancy as prospective predictors of alcohol and marijuana use. *Journal of Abnormal Psychology, 106*, 61-73.

Strecher, V. J., McClure, J., Alexander, G., Chakraborty, B., Nair, V., Konkel, J., et al. (2008). The role of engagement in a tailored web-based smoking cessation program: randomized controlled trial. *J Med Internet Res, 10*(5), e36.

Velicer, W. F., DiClemente, C. C., Prochaska, J. O., & Brandenburg, N. (1985). Decisional balance measure for assessing and predicting smoking status. *Journal of Personality and Social Psychology, 48*(5), 1279-1289.

Walters, S. T., Miller, E., Chiauzzi, E. (2005). Wired for wellness: e-interventions for addressing college drinking. *Journal of Substance Abuse Treatment, 29*(2), 139-45.

Webb, T. L., Joseph, J., Yardley, L., & Michie, S. (2010). Using the Internet to promote health behavior change: A systematic review and meta-analysis of the impact of theoretical basis, use of behavior change techniques, and mode of delivery on efficacy. *Journal of Medical Internet Research, 12*, e4.

Weinert, F., & Perlmutter, M. (1988). Memory development. Hillsdale, NJ: Lawerence Erlbaum

Weitzel, J. A., Bernhardt, J. M., Usdan, S., Mays, D., & Glanz, K. (2007). Using wireless handheld computers and tailored text messaging to reduce negative consequences of drinking alcohol. *J Stud Alcohol Drugs, 68*(4), 534-537.

White, A., Kavanagh, D., Stallman, H., Klein, B., Kay-Lambkin, F., Proudfoot, J., Drennan, J., Connor, J., Baker, A., Hines, E., & Young, R. (2010). Online alcohol interventions: A systematic review*. J Med Internet Res.,12*(5), e62.

Whittaker, R., Maddison, R., McRobbie, H., Bullen, C., Denny, S., Dorey, E., Ellis-Pegler, M., van Rooyen, J., & Rodgers, A. (2008). A multimedia mobile phone-based youth smoking cessation intervention: Findings from content development and piloting studies. *J Med Internet Res, 10*, e49.

World Health Organization (2004). Global status report on alcohol. *WHO Global Status Report on Alcohol 2004*, 1-88.
